# An Account of the Good Effects of Alkaline Salts in Counteracting the Poison of Corrosive Sublimate

**Published:** 1800-06

**Authors:** J. Evans

**Affiliations:** Ketley


### *An Account of the good Effects of Alkaline Salts in counteracting the Poison of Corrosive Sublimate.*

APRIL 17, 1796, John Podmore, a healthy young man, about 25 years of age, the son of a farmer in this neighbourhood, had an eruption upon his skin that was suspected to be the itch; in consequence of which, he was advised by his sister to buy three pennyworth of corrosive sublimate, (hydrarg. mur.) and two ounces of Glauber salts. The sublimate was to be dissolved in water, and applied as a lotion to the parts affected, and the salts to be taken in the morning fasting. The young man being totally unacquainted with the articles he had purchased, made use of the mercury instead of the salts, putting about one half of the quantity he was possessed of into a large cupful of warm water, stirring it about for some time with a spoon, and then drank it. Fortunately for him, a considerable portion of the sublimate remained undissolved in the bottom of the cup. The quantity swallowed, (from what was left) I supposed to have been not less than half a drachm. After this he took a walk into the fields, where finding himself seized with a violent sickness, vomiting and griping, it was with some difficulty he returned home. The family observing him so extremely ill, were much alarmed, which induced his mother to examine the shelf on which the salts and sublimate had been deposited, when, to her great concern, she discovered the mistake he had made; upon which I was desired to visit him as soon as possible, it being then near two hours after he had taken the poison. The moment I was informed of the young man's unfortunate situation, two cases of the efficacy of alkaline salts, administered on a similar occasion, published in the sixth vol. of the Edinburgh Med. Com. occurred to me. I immediately directed half an ounce of salt of tartar to be dissolved in eight ounces of water; of this solution he took a large table spoonful, which afforded him much relief, by abating for a short time the violent pain in his stomach; he afterwards took an emetic, which operated powerfully. Notwithstanding which, he had frequent returns of the pain, which extended through the whole course of the intestinal canal; but was as often relieved by the alkaline solution, which he took occasionally for several days. On the 18th, a purging draught was administered, and at night an opiate. This day his tongue began to swell; a cold sweat broke out all over him, accompanied with a slow and feeble pulse; a trembling of his limbs, and a total debility of the whole whole ſyſtem. 20th, Continued nearly the ſame, the opiate having procured but little reſt: ſeveral large puſtules of a livid hue appeared upon his face and breaſt. 21ſt, A copious ptyaliſm came on, which continued for many days. The inteſtinal canal was affected quite to the anus, from which oozed a corroſive ichor, that excoriated the external parts, and rendered them ſo ſore, that it was with difficulty he bore clyſters of olive oil and milk to be injected, which I directed to be adminiſtered twice a day, in order to lubricate and waſh the parts from the acrimonious diſcharge, as well as to empty the inteſtines of any fæces that might be retained in them. 23d, The pain of the ſtomach and bowels being conſiderably abated, I ordered him a decoction of Peruvian bark, with the addition of Huxham's tinct. of the ſame, and Chalybeate wine, to be taken three times a day. This plan was purſued till the 29th, taking opiates every night; but they did not afford him that relief I expected, for he got but little reſt until the ſalivation left him, which happened on the 28th. From that day his appetite began to mend, and his ſtrength gradually returning, he diſcontinued his medicines.

During the whole of his illneſs he was ſupported with milk, gruel, and chicken broth. Notwithſtanding which, he was more reduced both in bulk and ſtrength, than any patient I ever ſaw in ſo ſhort a ſpace of time. Had the alkaline ſolution been adminiſtered immediately after the poiſon was taken into the ſtomach, there is little doubt but the decompoſition of it would have been more effectual, and the patient's ſufferings in conſequence of it conſiderably allevated.

*Ketley, May 7, 1800.* J. EVANS, M. D.

